# MRI characteristics of lumbosacral dural arteriovenous fistulas

**DOI:** 10.3389/fneur.2023.1157902

**Published:** 2023-04-28

**Authors:** Jinyu Zhu, Wangshu Zhu, Minghua Li, Xiaoer Wei, Zheyi Chen, Yuehua Li

**Affiliations:** ^1^Institute of Diagnostic and Interventional Radiology, Shanghai Sixth People’s Hospital Affiliated to Shanghai Jiao Tong University School of Medicine, Shanghai, China; ^2^Department of Radiology, Shanghai Municipal Eighth People’s Hospital, Shanghai, China

**Keywords:** spinal dural arteriovenous fistula, lumbosacral region, MRI, time-resolved contrast-enhanced angiography, filum terminale vein

## Abstract

**Background and purpose:**

Spinal dural arteriovenous fistulas located in the lumbosacral region are rare and present with nonspecific clinical signs. The purpose of this study was to find out the specific radiologic features of these fistulas.

**Methods:**

We retrospectively reviewed the clinical and radiological data of 38 patients diagnosed with lumbosacral spinal dural arteriovenous fistulas in our institution from September 2016 to September 2021. All patients underwent time-resolved contrast-enhanced three-dimensional MRA and DSA examinations, and were treated with either endovascular or neurosurgical strategies.

**Results:**

Most of the patients (89.5%) had motor or sensory disorders in both lower limbs as the first symptoms. On MRA, the dilated filum terminale vein or radicular vein was seen in 23/30 (76.7%) patients with lumbar spinal dural arteriovenous fistulas and 8/8 (100%) patients with sacral spinal dural arteriovenous fistulas. T2W intramedullary abnormally high signal intensity areas were found in all lumbosacral spinal dural arteriovenous fistula patients, with involvement of the conus present in 35/38 (92.1%) patients. The “missing piece sign” in the intramedullary enhancement area was seen in 29/38 (76.3%) patients.

**Conclusion:**

Dilatation of the filum terminale vein or radicular vein is powerful evidence for diagnosis of lumbosacral spinal dural arteriovenous fistulas, especially for sacral spinal dural arteriovenous fistulas. T2W intramedullary hyperintensity in the thoracic spinal cord and conus, and the missing-piece sign could be indicative of lumbosacral spinal dural arteriovenous fistula.

## Introduction

Spinal dural arteriovenous fistula (SDAVF) is the most common type of spinal vascular malformation, constituting approximately 60–80% of all spinal arteriovenous malformations (1). The prevalence of SDAVF in the general population is 5–10 per million (2). The most common site of these fistulas is the thoracic spine, followed by the cervical and lumbosacral spine (1, 3). SDAVF located in the lumbosacral region (lsSDAVF) is rarer than SDAVF located in the other regions. Patients with lsSDAVF usually present with progressive paraparesis, often accompanied by gait and urinary disturbances due to congestive myelopathy (4, 5). The clinical presentation is nonspecific, and the condition is therefore easily confused with other diseases such as protrusion of intervertebral disk (6, 7). Poor awareness of the imaging features leads to misdiagnosis and delayed or incorrect treatment (8) with resulting severe permanent morbidity.

No large series on lsSDVAF has been published so far. In this retrospective study, we review the clinical characteristics and radiologic features of patients diagnosed with lsSDAVF in our institute.

## Materials and methods

We retrospectively reviewed the records of all patients diagnosed with lsSDAF in our institution from September 1, 2016, to September 30, 2021. Most of the patients were referred to our hospital after failure to arrive at a diagnosis. All patients underwent MRI and DSA examinations, and were treated with either endovascular or neurosurgical strategies at our hospital after definite diagnosis based on DSA findings. After discharge, the patients were followed up by outpatient visit or by regular phone calls.

The clinical records of the patients were searched to collect data on demographics, clinical and neurologic presentations, treatment, and complications. The data were recorded using a standardized case report form. Neurologic function was graded according to the mRS (9).

### MR imaging/MRA

MR examinations were performed on a 3.0 T MRI system (MAGENTOM Verio, Siemens Healthcare, Erlangen, Germany). The MR sequences included sagittal T1-and T2-weighted, and axial T2-weighted, time-resolved contrast-enhanced three-dimensional (3D) MRA, including time-resolved angiography with stochastic trajectories (TWIST), and sagittal and coronal 3D-T1-volumetric interpolated breath-hold examination (3D-T1-VIBE). For MRA, the contrast agent (0.5 mL/kg of 0.5 mmol/mL gadodiamide) was injected as a test bolus through the antecubital vein at a rate of 3–4 mL/s with a high-pressure syringe. Fat-suppressed T2-weighted fast spin-echo sequence was acquired to visualize the anatomy of the spine and localize the range of lesions in the spinal cord. The VIBE images showed the perimedullary surface veins and enhancement pattern of the spinal cord. TWIST helped differentiate between spinal artery and vein. Deformed blood vessels were displayed on the coronal plane by using curved planar reformation and MIP. The MRA data were independently analyzed by two neuroradiologists (with 35 and 10 years’ experience of neuroimaging); disagreements were resolved by discussion until consensus was reached.

### DSA

For all patients, DSA was performed with a bipalanar unit (Artis zee; Siemens Healthcare, Forchheim, Germany) within a month after MRI examination to identify the feeding artery, the location of fistula, and the direction of the draining vein. The images were evaluated by three observers with 35, 18, and 10 years’ experience in the field of neuroradiology.

### Protocol approval, registration, and patient consent

Approval was obtained from the Human Studies Committee of Shanghai Sixth People’s Hospital Affiliated to Shanghai Jiao Tong University School of Medicine prior to initiation of the study. Written informed consent was obtained from each participant before enrollment in the study.

## Results

### Clinical characteristics

The 38 patients [23 (60.5%) males] had median age of 66 years (range, 37–79 years). While 30 patients had SDAVF in the lumbar spine, eight had SDAVF in the sacral region. The median duration of symptoms (from onset to diagnosis) was 5 months (range, 1–120 months). Most patients had either been misdiagnosed with other neurological diseases at other hospitals or the location of fistula had not been identified. While 11 patients had hypertension, eight had diabetes. The majority of patients (34/38, 89.5%) had sensory or motor disorders of both lower limbs as the first symptoms. At admission to our hospital, 34 patients had difficulty in walking on both lower limbs, eight had lower limb paralysis, four had low back pain, and 34 had numbness or pain in both lower limbs; 30 patients suffered from urination and defecation disturbance. Among the 38 patients, most had mRS score of 3 or 4 (17 had score of 3 while 11 had score of 4), and only two patients had mRS score of 5. All the clinical features of lumbosacral SDAVF patients are listed in Table 1.

**Table 1:** Demographic and clinical features of lumbosacral SDAVF patients (*n* = 38).

Characteristics	Data
Median age, years (range)	66 (37–79)
Sex	
Male	23
Female	15
Comorbidities	
Hypertension	11/38
Diabetes	8/38
Clinical features	
Lower extremity weakness	34/38
Low back pain	4/38
Sensory disturbance	34/38
Urination and defecation disturbance	30/38
mRS score	
1 point	2
2 points	6
3 points	17
4 points	11
5 points	2

### Radiologic findings

**Figure 1:**
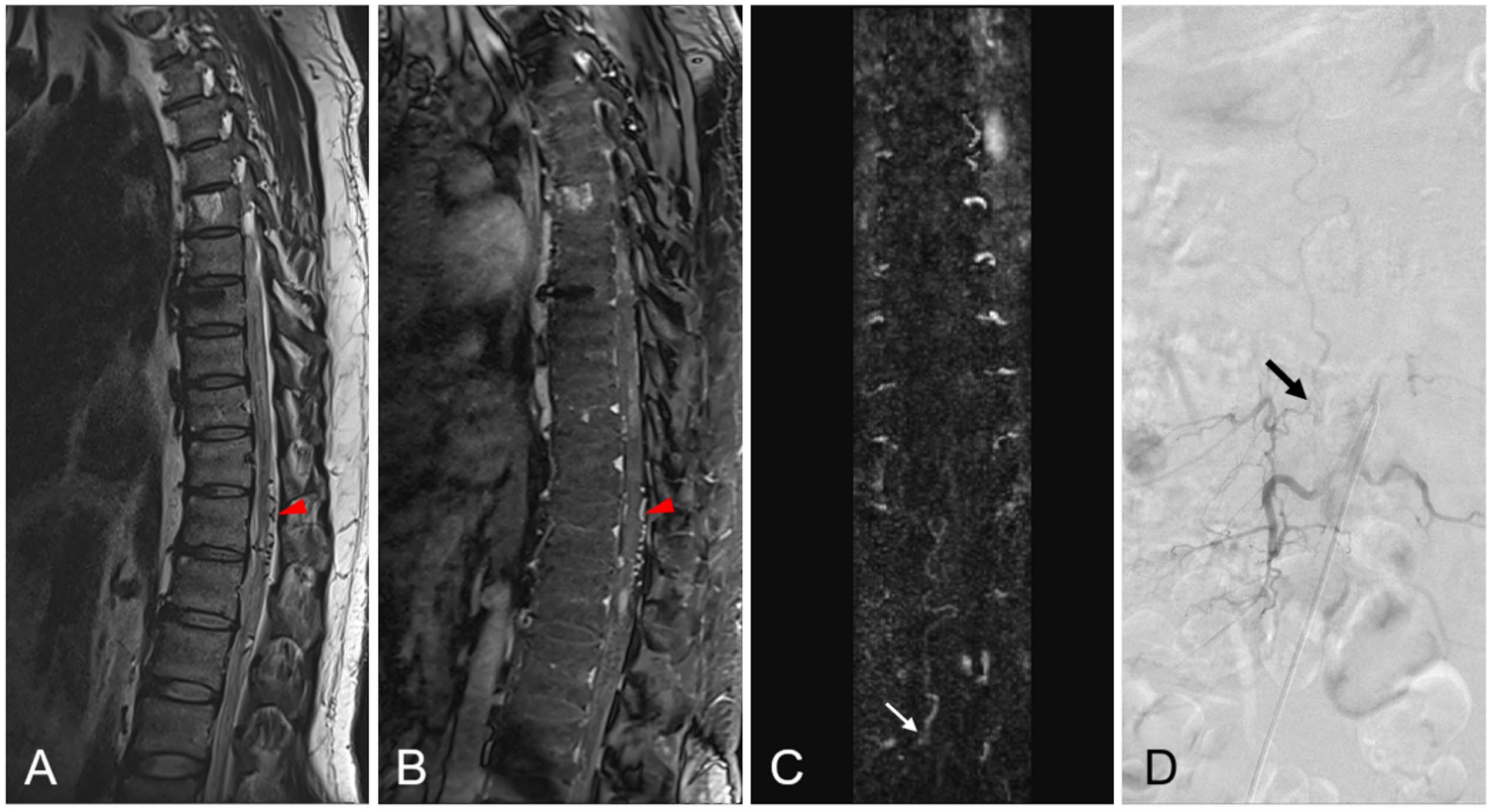
A 42-year-old patient with spinal dural arteriovenous fistula (SDAVF) at the L2-3 level on the right side. **(A,B)** Sagittal T2-weighted image and enhanced T1-weighted image shows prominent perimedullary vein (*red arrowhead*). **(C)** The coronal MRA MIP image demonstrates the feeding artery of the SDAVF at the L2-3 level on the right side (*white arrow*). **(D)** The localization of this origin is confirmed by DSA (*black arrow*).

**Figure 2:**
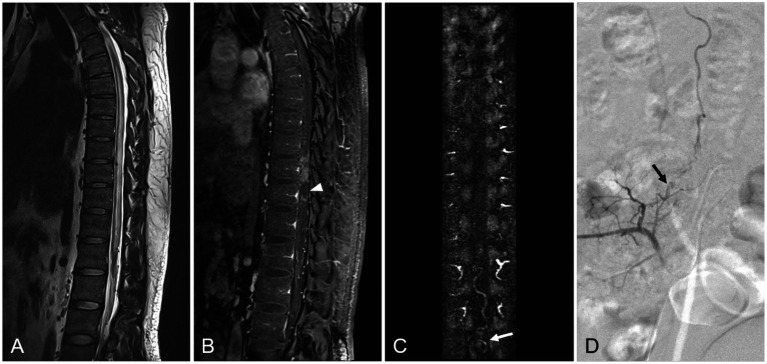
A 70-year-old patient with sacral SDAVF. **(A)** Sagittal T2-weighted image reveals a signal intensity increase of spinal cord, involving the conus. **(B)** The enhanced T1-weighted image demonstrates the patch unenhanced region in the spinal cord (*white arrowhead*). **(C)** In the coronal MRA MIP image, the dilated filum terminale vein suggests that the feeding artery is probably located in the sacrum (*white arrow*). **(D)** DSA examinations confirm that the fistula supplied via the right internal iliac artery (*black arrow*).

On preoperative MR imaging, T2W intramedullary abnormally high signal intensity areas were found in all 38 patients. These high signal areas involved a mean of 6.5 vertebrae (range, 3–10.5 vertebrae). The T2W high signal intensity involved the conus in 35/38 (92.1%) patients. Pretreatment gadolinium-enhanced MRI showed intraparenchymal enhancement in all 38 patients. Within the intraparenchymal enhancement, 29 of the 38 patients (76.3%) displayed a characteristic unenhanced patchy area, which we termed the “missing-piece sign.” In the 30 lumbar SDAVF patients, the feeding artery was located on the left in 13 (43.3%) patients and on the right in 17 (56.7%) patients. In all eight sacral SDAVF patients, the feeding vessels were the internal iliac vessels. The dilated filum terminale vein (FTV) or radicular vein was found in 23/30 (76.7%) lumbar SDAVF patients and in 8/8 (100%) sacral SDAVF patients. The radiologic features of lumbosacral SDAVF are showed in Table 2 and Figures 1, 2.

**Table 2:** Radiologic features of lumbosacral SDAVF.

Characteristic	Data
Spinal cord edema	38/38
Intramedullary abnormal hyperintensity regions (vertebral levels)	6.5
T2W high signal intensity involving conus	35/38
Missing-piece sign	29/38
Shunt location	
Lumbar	30
Left	13/30
Right	17/30
Sacral	8
Dilated FTV or radicular vein	31/38
Lumbar	23/30
Sacral	8/8

## Discussion

Spinal dural arteriovenous fistula in the lumbosacral region is rare, especially so in the sacral region (only ~4% being located in this region) (4). In this study, we tried to identify the characteristic clinical and radiological manifestations of lumbosacral dural arteriovenous fistulas, the aim being to increase awareness of the features of this rare condition and thereby enable early diagnosis. We found that presence of serpentine tortuous vessels in the lumbosacral spinal canal is a specific manifestation of lumbosacral dural arteriovenous fistula. Abnormally high intramedullary T2W signal and the missing-piece sign are other imaging features indicative of this condition.

The clinical manifestations of lsSDAVF are similar to those of SDAVF in other regions and other various myelopathy disorders (10, 11). Most of our patients had motor or sensory disorders in both lower limbs as the first symptom, with some patients also having urination and defecation disturbance. In all patients, the symptoms slowly progressed gradually over time. Nevertheless, the similar neurological symptoms caused a high rate of delayed diagnosis and consequently late treatment.

Jablawi et al. (12) demonstrated that shorter duration of symptoms before treatment (<6 months) was associated with better long-term outcome, and that age and neurologic status had no influence on long-term outcome. Therefore, it is necessary to make a clear diagnosis and to identify the location of the lesion as soon as possible.

Some specific features in MR imaging can help in diagnosis of lsSDAVF. The dilated FTV or radicular vein was shown in all eight patients with sacral SDAVF and in 23 of 30 patients with lumbar SDAVF in our study. The FTV, which is located in front of the terminal filament, is continuous with the anterior spinal vein and plays a major role in intradural drainage. The FTV is the only intradural vein and longitudinal draining vein below the level of the L2 vertebral body (13, 14). Any arteriovenous shunt of the sacral SDAVF draining toward the spinal cord must drain through the FTV (14, 15). Our MRI findings confirm that a prominent arterialized filum terminale vein or radicular vein, even if there are no prominent perimedullary vein, is strong evidence in support of the presence of lsSDAVF, especially sacral SDAVF.

Intramedullary high signal intensity in T2W images is a known imaging manifestation of SDAVF (16) and, in ~80% of cases, this involves the conus (17). In our study, all 38 patients (100%) showed spinal cord edema, involving a mean of 6.5 vertebrae; in 35 of the 38 patients, the edema involved the conus. This is consistent with previous reports. Some studies have shown that the range of intramedullary T2W high signal intensity is related to the symptoms and prognosis of patients (18). We could not verify this finding in our small sample.

In most of the enhanced TWIST images, we found patchy unenhanced areas in the enhanced spinal cord; we termed this the “missing-piece sign.” In our study, 29 (76.3%) patients displayed this missing piece sign. Spinal cord enhancement indicates contrast infiltration due to disruption of the local vascular barrier, secondary to progressive venous hypertension in SDAVF (19). The missing-piece sign may indicate that the spinal cord vascular barrier has not been destroyed and, therefore, may be associated with prognosis. Zalewski et al. claimed that this sign is specific for SDAVF and can be used to differentiate it from other spinal cord lesions (20). However, no evidence can be used to prove the direct relationship between missing-piece sign and lsSDAVF. Further research is needed to verify this possibility.

Time-resolved contrast-enhanced MRA might improve the accuracy of SDAVF diagnosis. With further improvements in time resolution, it might help in precisely locating the fistula before operation or interventional embolization treatment. In our study, the location of the fistula was accurately identified by MR imaging in all 30 lumbar SDAVF patients and confirmed by DSA. Because of the limitation of FOV, the location of some sacral SDAVF could not be clearly identified; however, in all patients, MRA images suggested that the feeding artery might originate from the sacral region.

Our study has some limitations. First, for some patients, MRA could not be performed due to the contraindications of MRI, and the CTA examination was chosen instead. Further studies are required to demonstrate the CTA findings of lsSDAVF. Second, our study population of sacral SDAVF was relatively small. More sample capacities of sacral SDAVF are needed to improve the generalizability of our results.

## Conclusion

Lumbosacral spinal dural arteriovenous fistula presents with nonspecific clinical features, which makes it difficult to diagnose. Dilatation of the filum terminale vein or radicular vein, T2W hyperintensity in the thoracic spinal cord and conus, and the missing-piece sign could be imaging features which would help in early diagnosis of this rare condition. The presence of the dilated FTV or radicular vein is strongly indicative of lumbosacral spinal dural arteriovenous fistula, especially sacral dural arteriovenous fistula.

## Data availability statement

The original contributions presented in the study are included in the article/supplementary material, further inquiries can be directed to the corresponding author.

## Ethics statement

Written informed consent was obtained from the individual(s) for the publication of any potentially identifiable images or data included in this article.

## Author contributions

JZ and YL conceived and designed of the study. WZ, XW, and ZC organized the database and performed the MRI scan. DSA was performed by ML. JZ wrote the first draft of the manuscript. YL and ML reviewed and revised the manuscript. All authors contributed to the article and approved the submitted version.

## Funding

This study was supported by the National Key Research and Development Program of China (Grant No. 2019YFC0117703); the National Natural Science Foundation of China (Grant No. 8225024); and Shanghai Key Clinical Specialty (Grant No. shslczdzk03203).

## Conflict of interest

The authors declare that the research was conducted in the absence of any commercial or financial relationships that could be construed as a potential conflict of interest.

## Publisher’s note

All claims expressed in this article are solely those of the authors and do not necessarily represent those of their affiliated organizations, or those of the publisher, the editors and the reviewers. Any product that may be evaluated in this article, or claim that may be made by its manufacturer, is not guaranteed or endorsed by the publisher.
